# Effects of traditional brewery dried residue and field pea hull mixtures supplementation on feed utilization and performance of Washera sheep fed natural pasture grass hay as basal diet

**DOI:** 10.1002/vms3.1226

**Published:** 2023-07-28

**Authors:** Shashie Ayele Yimenu, Ayalew Abebe

**Affiliations:** ^1^ Department of Animal Science Bahir Dar University Bahir Dar Ethiopia; ^2^ Department of Animal Science Bonga University Bonga Ethiopia; ^3^ Department of Animal Science Debre Markos University Debre Markos Ethiopia

**Keywords:** digestibility, feed intake, non‐conventional supplement, small ruminant

## Abstract

**Background:**

The major feedstuffs (natural pasture and crop residues) used for sheep in Ethiopia are fibrous and the crude protein (CP) content is less than 7% that is inadequate to meet the maintenance requirement of sheep. These poor quality feeds should be improved and can be improved through supplementation with nutritious feedstuff. Therefore, to overcome this challenge, there is a need to look for some alternatives but locally available and cheap sources of protein. In this regard, traditional brewery dried residue (TBDR) and field pea hull (FPH) could be an important sources of feed for ruminant livestock.

**Objectives:**

This experiment was carried out to evaluate the effects of supplementing mixtures of TBDR and FPH on the feed intake, digestibility, live weight gain, and economic feasibility of the feeding treatments.

**Methods:**

In a 3‐month experiment, 20 yearling intact male Washera sheep were blocked based on their initial BW of 22.1 ± 1.58 kg (mean ± standard error of mean), and treatment diets were randomly assigned within a block. Treatments comprised feeding natural pasture grass hay (NPGH) ad libitum + 50 g ground nut cake (GNC) (*T*_1_, control); *T*_1_ + supplemented with 25% TBDR:75% FPH (*T*_2_); *T*_1_ + 50% TBDR:50% FPH (*T*_3_) and *T*_1_ + 75% TBDR:25% FPH (*T*_4_). The supplement feed was offered twice a day at 08:00 and 16:00 while, common salt lick and water were available all time.

**Results:**

NPGH, FPH, TBDR, and GNC in the current study contained 5.7%, 13.4%, 22.2%, and 45.4% CP and 62%, 61%, 34%, and 20% neutral detergent fibre, respectively. Sheep in supplemented treatments had higher (*p* < 0.05) apparent digestibility percentage of dry matter and nutrients than those in *T*_1_. Among the supplemented treatments, sheep fed *T*_4_ recorded 4.49 kg extra (*p* < 0.0001) body weight as compared to that fed *T*_1_ by growing at an average of 49.8 g/day and returned the highest net income (255.8 Ethiopian birr)

**Conclusion:**

Thus, based on the highest body weight gain and net return, the combinations of 75% TBDR and 25% FPH (*T*_4_) appeared to be recommendable for poor farmers.

## Graphical abstract

Due to higher weight gain and profitability of the feeding regime, *T*_4_ is recommended for small scale sheep fattener who live in areas where there is shortage of concentrate supplement.

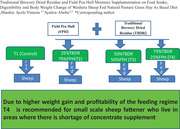

## 1 INTRODUCTION

Sheep contribute much to the livelihood of farmers and pastoralists all over the world by providing meat, milk, fibre, skin, manure, and immediate cash income to their owners. Sheep in Ethiopia are also among the most economically important livestock species in the country, playing a crucial role in the livelihood of smallholder farmers and pastoralist (Nigussie et al., 2013; Zewdu et al., 2009). However, sheep, in tropical countries in general andin Ethiopia in particular, are managed under low input extensive production systems and consequently produced the lowest carcass weight compared to the world and other least developed African countries (Ayele & Urge, 2019).

About 93% of the feed stuff used for sheep in Ethiopia are coming from natural pasture and the residues after grain threshing (CSA (Central Statistical Agency), 2021), such feed resources mostly contain inadequate crude protein (CP) (less than 7%) to meet the minimum requirement for growth of rumen microorganisms (Van Soest, 1994) and consequently low digestibility, and thus have limited voluntary intake by animals. Therefore, these poor‐quality feed resources have to improve and can be improved through supplementation with conventional agro‐industrial by‐products such as flour milling by‐products (brans) and residues from oil extraction factories (Ayele et al., 2017). However, using conventional agro‐industrial by‐products is unaffordable and inaccessible to rural farmers (Mekasha et al., 2002). Therefore, to overcome this challenge, there is a need to look for some alternative but easily available and cheap sources of protein. In this regard, non‐conventional feedstuffs could be used as alternative supplements in sheep production. Among such supplements, traditional brewery dried residue (TBDR), and field pea (*Pisum sativum*) hull could be important sources of feed for ruminant livestock.

A legume grain crop, field pea, is used all over the world in human diets (McKay et al., 2003), and the hull used as livestock feed. In Ethiopia, field pea is an important pulse crop next to haricot beans and chickpeas (CSA (Central Statistical Agency), 2021). Field pea is widely planted and used in Amhara, Oromia, Central, Western, and Southern Tigray of Ethiopian regions (CSA (Central Statistical Agency), 2021). TBDR (dried Atella) is the byproduct of cereal grain fermentation residues from alcoholic drinks and beverages such as Tella. These two feedstuffs are available easily as energy and protein feed sources that create an opportunity for a limited period planned fattening of small ruminants in areas where feed shortage in quality and quantity is available. In addition, there is a growing trend in the expansion of smallholder fattening practices in urban and pre‐urban areas of the country to ensure household food security and to alleviate poverty as well as to supply better quality animals to satisfy the increasing demand in the domestic market as well as to export live sheep and child meat to many Middle East countries ethiopian sheep & goat productivity improvement program (ESGPIP), 2011; Food and agricultural organization (FAO), 2019).

The feeding trial studies using either of one as a supplement for sheep have been studied and reported improvements in feed conversion efficiency and weight gain in sheep (Ayenew et al., 2012; Mekasha et al., 2002; Mekcha et al., 2019; Mesganaw, 2014). However, there is a gap in information for the use of these supplements in combination (TBDR + FPH) and particularly concerning the ratio at which TBDR and FPH could be mixed to prepare a supplement ration that will improve the weight gain of sheep in the traditional livestock production systems. Therefore, this study was designed to evaluate the effects of TBDR and field pea hull (FPH) mixtures supplementation on feed intake, digestibility, body weight change and economic feasibility of Washera sheep fed natural pasture grass hay (NPGH) as basal diet.

## 2 MATERIALS AND METHODS

### 2.1 Study area description

The experiment was conducted in Hulet‐Eju Enessie district, Keranio Keble, 11° 15′N latitude and 37° 45′E longitude at an altitude of 1200 m above sea level, East Gojjam Zone, Amhara Regional State, Ethiopia. The area receives 1200 mm average annual rainfall of bimodal type that occurs between March–April and June–August. The average annual temperature ranges from 8 to 25°C (Derejaw et al., 2013).

### 2.2 Experimental sheep management

Intact, male, 20 yearling, Washera sheep with an initial body weight (IBW) of 22.11 ± 1.58 kg (mean ± standard error of mean) were purchased from Woyin‐wuha local market. The age of the animals was determined by observing the size of the incisor teeth (Ethiopia Sheep and Goat Productivity Improvement Program [ESGPIP, 2009) and information obtained from the owners. The sheep were dewormed with Albendazole (15 mg/kg) and sprayed with Diazinine (1 mL/L of water) against common internal and external parasites, respectively, and were vaccinated against anthrax (0.5 mL/head), sheep Pox (1 mL/head) and pasturolosis (2 mL/head) during the quarantine period of 21 days. Thereafter, sheep were penned (136 cm × 80 cm, pen area) individually in a well‐ventilated house that was equipped with feeding (separate for supplement and hay) and watering troughs. Each sheep was tagged with neck collars for identification and ease of record keeping.

### 2.3 Feed collection and preparation

NPGH, composed of mainly *Cynodon dactylon*, *Eragrostis*, *Sporobolus* and *Pennisetum* species, was purchased from surrounding farmers’ fields, harvested at the proper stage, dried, transported to the study site, and stored under shade to maintain its quality. NPGH was manually chopped to the size of about 4–5 cm (Likawent et al., 2012; Panadi et al., 2021) for better utilization and used as a basal diet throughout the experimental period.

Wet traditional brewer residue, a byproduct of Tella (local drink), was collected from households who brew it regularly using ingredients such as maize, barley, and wheat for the preparation. The wet traditional brewery residue was dried on a canvas to reduce the moisture content at the required level (15%). To ensure uniform drying and to avoid clumping, wet, traditional brewery residue was spread thinly on the canvas and mixed with a fork several times a day. All the required quantities were collected before the commencement of the experiment, thoroughly mixed, and stored in the well‐ventilated house. The FPH was collected from a nearby agro‐industry mill. A groundnut cake was bought from the surrounding oil extraction factory.

### 2.4 Experimental design, treatments, feeding and measurements

The experiment was laid out in a randomized complete block design based on their IBW and stratified into five blocks consisting of four animals per block, and treatment feeds was assigned randomly to each animal within a block. Supplement feed (450 g/day dry matter [DM]) was offered twice a day at 08:00 and 16:00 with a common salt lick and water offered free choice. The treatments used were feeding NPGH ad libitum + 50 g ground nut cake (GNC) (*T*_1_, control); *T*_1_ + supplemented with 25% TBDR:75% FPH (*T*_2_); *T*_1_ + 50% TBDR:50% FPH (*T*_3_) and *T*_1_ + 75% TBDR:25% FPH (*T*_4_). The basal diet was provided to all animal ad libitum to ensure a refusal of a 20% allowance of the previous days’ intake, and the intake was adjusted once every third day. Fifty grams of GNC were added to all animals to meet the maintenance requirement of the sheep in the control group. Data collection was started after 15 days of experimental feeds and pen adaptation. Feeds offered and the corresponding refusals were recorded daily to determine the daily feed intake for each animal for the duration of the experiment (90 days). Samples of feeds offered and refusals were taken every day and pooled for each feed offer and per treatment for the refusals. Thereafter, at the end of the experiment, pooled feed and refusal samples were thoroughly mixed and sub‐sampled for chemical analysis.

Metabolized energy (ME) MJ/day intake was estimated from digestible organic matter intake (DOMI) values by using the equation of AFRC (Agricultural Food and Research Council) (1993), where DOMI is digestible organic matter intake (in grams per kilogram DM). The IBWs of the experimental animals were determined by taking the mean of two consecutive weights measured after overnight fasting at the beginning of the feeding trial. Thereafter, animals were weighed individually every 10 days throughout the feeding trial period of 90 days. Average daily gain was calculated as a difference between final and initial BW divided by the number of feeding days. Feed conversion efficiency was calculated by dividing the daily BW change to daily feed intake.

A digestibility trial was conducted after the completion of the feeding trial using all sheep. Sheep were harnessed with faecal collecting bags to collect faeces for digestibility determination. Sheep were allowed to acclimatize to the faecal collecting bags for 3 days, and this was followed by a collection of faeces for 7 days. The collected faeces were weighed daily and 20% of the daily faeces voided by each animal was sampled and pooled over the collection period for each sheep separately and stored in a deep freezer at −20°C using plastic bags. At the end of the digestibility trial, the collected faecal samples were thoroughly mixed, then 10% of the total collected sample from each animal was sub‐sampled, weighed, partially dried at 60°C for 72 h, ground, and stored in airtight polyethylene plastic bags pending chemical analysis. During the digestibility period, hay offered and refused was recorded daily and samples from feed offered and refusals from each animal were taken daily to make a composite sample. The apparent digestibility percentage of DM was calculated as DM intake minus DM in faeces divided by DM intake and times by a hundred. Likewise, the apparent digestibility percentage of other nutrient intakes (organic matter [OM], CP, neutral detergent fibre [NDF], and acid detergent fibre [ADF]) was calculated as nutrient intake minus nutrients in faeces divided by nutrient intake and times by hundred (McDonald et al., 2010).

### 2.5 Partial budget analysis

The analysis involved the calculation of the variable costs of sheep, feeds and benefits gained from the result (Upton, 1979) to evaluate the economic profitability of the treatment feeds. Purchasing and selling prices of sheep, purchasing price of hay, FPH, and traditional dried brewery residue were recorded. Other expenses such as feed and sheep transportation costs, mineral licks, housing, labour, and veterinary services, which were common for all treatments, were not considered in the calculation of the partial budget analysis. The selling price of each sheep was determined by inviting well‐experienced three sheep dealers, who know the prevailing market price of sheep in the study area's market. Therefore, the average price determined by the dealers was used as the selling price of the sheep.

The cost of feeds was computed by multiplying the actual DM intake of feed for the whole feeding period (90 days) with the current purchase price of each treatment feed. The total cost associated with each sheep during the experimental period in each treatment was summed and the average was taken as a total variable cost (TVC). The total return (TR) was calculated as the difference between the selling and purchasing prices of the experimental animals. The net income (NI) was calculated as the amount of money left when TVC is subtracted from the TR. The change in NI (ΔNI) was calculated as the difference between the change in TR (ΔTR) and the change in TVCs (ΔTVC). The marginal rate of return (MRR) measures the increase in NI (ΔNI) associated with each additional unit of expenditure (ΔTVC) and expressed in percentage as$\;\mathrm{MRR}\;=\frac{\Delta \mathrm{NI}}{\Delta \mathrm{TVC}}\;\times \;100$.

### 2.6 Chemical analysis

Faeces and feed samples (offer and refuse) were dried at 105°C overnight in a forced draft oven for DM determination. The remaining feed and faeces samples were dried at 60°C to constant weight and ground through a 1 mm sieve screen for DM, total ash and nitrogen determination following the procedure of AOAC (Association of Official Analytical Chemists) (1990). The nitrogen content of the samples was determined using Kjeldahl procedure, and then CP was determined as N × 6.25. NDF and ADF were determined following the procedures described by Van Soest and Robertson (1985). OM was calculated as ash deducted from hundreds.

### 2.7 Statistical analysis

Data obtained on feed intake, digestibility and body weight change were subjected to the analysis of variance using PROC GLM procedure of SAS software package version 9.1 (SAS (Statistical Analysis Systems Institute), 2008). Least square means and standard errors were determined using the LSMEANS and STDERR statements in PROCGLM. Mean separations were determined using Tukey's multiple comparison procedure. The statistical model used was *Y_ij_* = *μ* + *T_i_* + *B_j_* + *E_ij_*, where *Y_ij_* is the response variable, *μ* is the overall mean, *T_i_* is the treatment effect, *B_j_* is the block effect and *E_ij_* is the random error.

## 3 RESULTS

### 3.1 Chemical composition of treatment feeds

The chemical composition of the treatment feeds is presented in Table 1. The CP content of NPGH used in the current study is low compared to the CP contents in TBDR and FPH. On the other hand, the NDF and ADF contents were higher in NPGH followed by FPH, whereas TBDR had the lowest NDF and ADF contents. The CP content of the refusals of the basal diet observed in the present study was lower as compared to the CP content of hay offered. Conversely, hay refusals in all treatments had relatively higher NDF and ADF content than the offered (Table 1).

**TABLE 1:** Chemical composition of feeds offered and hay refused (% dry matter [DM]).

	Chemical composition					
Feeds	DM (%)	OM (% DM)	CP (% DM)	NDF (% DM)	ADF (% DM)	ADL (% DM)
Feed offered						
Hay	90	92.3	5.7	62.2	44.4	12.2
TBDR	90	96.6	22.2	34.4	26.7	8.9
FPH	91	90.4	13.4	61	52.2	14.3
TBDR:FPH (25:75)	90.8	91.9	15.6	61.9	45.8	12.9
TBDR:FPH (50:50)	90.5	93.5	17.8	52.7	39.4	11.6
TBDR:FPH (75:25)	90.3	95	20	43.6	33	10.2
GNC	92	93.5	45.4	20	12.9	4.5
Refusal hay						
*T*_1_	90	93.4	3.4	63.2	44.4	11.1
*T*_2_	91	93.4	3.4	69.9	51	14.3
*T*_3_	91	92.3	3.9	66.5	48.8	13.3
*T*_4_	90	92.2	3.6	71.1	53.3	14.4

*Note*: *T*_1_: hay + 50 g GNC; *T*_2_: *T*_1_ + 25% TBDR + 75% FPH; *T*_3_: *T*_1_ + 50% TBDR + 50% FPH; *T*_4_: *T*_1_ + 75% TBDR + 25% FPH.

Abbreviations: ADF, acid detergent fibre; ADL, acid detergent lignin; CP, crude protein; FPH, Field pea hull; GNC, ground nut cake; NDF, neutral detergent fibre; OM, organic matter; TBDR, traditional brewery dried residue.

### 3.2 Feed intake

The basal diet (NPGH) intake was reduced (*p* < 0.0001) in supplemented sheep by about 207 g/day (*T*_2_), 220 g/day (*T*_3_) and 170 g/day (*T*_4_) than sheep consumed the control treatment (Table 2). On the other hand, supplementation improved (*p* < 0.0001) total DM intake by 280 g/day (*T*_4_), 243 g/day (*T*_2_) and 230 g/day (*T*_3_) over the control treatment. Supplementation increased (*p* < 0.0001) the intake of OM, CP, ME, NDF and ADF as compared to sheep in control group. The total average daily CP intake was higher (*p* < 0.001) in the order of *T*_4_ = *T*_3_ > *T*_2_ in supplemented groups, but there was no significant difference (*p* > 0.05) among supplemented groups of sheep in total DM intakes as percentage and metabolic body weights. Similarly, ME intakes were comparable for all supplemented groups. NDF and ADF intakes in sheep fed *T*_2_ supplement were higher (*p* < 0.0001) than those groups of sheep consumed *T*_3_ and *T*_4_.

**TABLE 2:** Daily feed intake of Washera sheep fed on natural pasture grass hay (NPGH) and supplemented with traditional brewery dried residue (TBDR) and field pea hull (FPH) mixtures.

	Treatments					
Intake (g/d)	*T*_1_	*T*_2_	*T*_3_	*T*_4_	SEM	*p* value
NPGH	655^a^	448^c^	435^c^	485^b^	29.78	<0.0001
Supplement DMI	50	500	500	500		
Total DM (g/d)	705^c^	948^b^	935^b^	985^a^	29.78	<0.0001
DM (%BW)	3.0^b^	3.7^a^	3.6^a^	3.8^a^	0.24	<0.0001
DM (g/kg BW^0.75^)	66.3^d^	83.66^a^	82.21^a^	86.58^a^	4.42	<0.0001
**Nutrient intake**						
Total CP	59.7^c^	118^b^	127.2^a^	135.4^a^	2.31	<0.0001
Total OM	650^c^	870^b^	874^b^	936^a^	27.88	<0.0001
Total NDF	417^c^	566^a^	518^b^	508^b^	18.53	<0.0001
Total ADF	297^c^	411^a^	377^b^	370^b^	13.23	<0.0001
ME (MJ/day)	6.7^b^	9.87^a^	10.3^a^	10.9^a^	0.31	<0.0001

*Note*: ^a,b,c,d^Different superscriptsn a row are significantly different at p<0.05;*T*_1_: hay + 50 g GNC; *T*_2_: *T*_1_ + 25% TBDR + 75% FPH; *T*_3_: *T*_1_ + 50% TBDR + 50% FPH; *T*_4_: *T*_1_ + 75% TBDR + 25% FPH.

Abbreviations: ADF, acid detergent fibre; ADL, acid detergent lignin; CP, crude protein; DM, dry matter; GNC, ground nut cake; ME, metabolized energy; NDF, neutral detergent fibre; OM, organic matter; SEM, standard error of mean.

### 3.3 Apparent dry matter and nutrient digestibility

Sheep in supplemented treatments had higher (*p* < 0.0001) DM, OM, CP, NDF and ADF apparent digestibility percentages than those in the control treatment (Table 3). Among the supplemented treatments, *T*_4_ achieved the best apparent digestibility of CP. No differences (*p* > 0.05) were evidenced in the apparent digestibility percentage of ADF and NDF among supplement treatments.

**TABLE 3:** Apparent dry matter and nutrients digestibility by Washera sheep fed on natural pasture grass hay supplemented with traditional brewery dried residue (TBDR) and field pea hull (FPH) mixtures.

	Treatments					
Apparent digestibility (%)	*T*_1_	*T*_2_	*T*_3_	*T*_4_	SEM	*p* value
DMD	62.9^d^	70.0^c^	72.6^a^	71.9^b^	3.39	<0.0001
OMD	64.6^d^	70.9^c^	73.9^a^	72.9^b^	3.35	<0.0001
CPD	55.6^d^	66.3^c^	69.43^b^	74.5^a^	4.90	<0.0001
NDFD	54.6^b^	62.0^a^	62.5^a^	64.5^a^	4.97	0.001
ADFD	52.57^b^	61.6^a^	62.3^a^	63.7^a^	5.43	0.020

*Note*: ^a,^ ^b,c,d^Different superscript in a row are signficantly different at p<0.05;*T*_1_: hay + 50 g GNC; *T*_2_: *T*_1_ + 25% TBDR + 75% FPH; *T*_3_: *T*_1_ + 50% TBDR + 50% FPH; *T*_4_: *T*_1_ + 75% TBDR + 25% FPH.

Abbreviations: ADFD, acid detergent fibre digestibility; CPD, crude protein digestibility; DMD, dry matter digestibility; GNC, ground nut cake; NDFD, neutral detergent fibre digestibility; OMD, organic matter digestibility; SEM, standard error of mean.

### 3.4 Body weight change and feed conversion efficiency

Supplemented sheep gained higher (*p* < 0.0001) FBW, ADG, and FCE than sheep fed control treatment. Among the supplemented groups of sheep, sheep fed *T*_4_ gained 4.49 kg extra body weight as compared to those fed control treatment by growing at an average of 49.8 g/day (Table 4).

**TABLE 4:** Body weight change, feed conversion efficiency and average daily gain of Washera sheep fed on natural pasture grass hay and supplemented with traditional brewery dried residue (TBDR) and field pea hull (FPH) mixtures.

Parameters	Treatments					
	*T*_1_	*T*_2_	*T*_3_	*T*_4_	SEM	*p* value
Initial body weight (kg)	21.6	21.9	22.7	22.2	1.58	0.7421
Final body weight (kg)	24.3^c^	27^b^	27.9^ab^	29.3^a^	1.62	0.0014
Body weight change (kg)	2.64^c^	5.08^b^	5.2^b^	7.1^a^	0.99	<0.0001
ADG (g)	29.3^c^	56.4^b^	57.8^b^	79.1^a^	11.05	<0.0001
FCE (g ADG/g DMI)	0.042^c^	0.059^b^	0.062^b^	0.08^a^	0.01	<0.0001

*Note*: a–c; means with different superscripts in a row are significantly different at *p* < 0.05. *T*_1_: hay + 50 g GNC; *T*_2_: *T*_1_ + 25% TBDR + 75% FPH; *T*_3_: *T*_1_ + 50% TBDR + 50% FPH; *T*_4_: *T*_1_ + 75% TBDR + 25% FPH.

Abbreviations: ADG, average daily gain; FCE, feed conversion efficiency; GNC, ground nut cake; SEM, standard error of mean.

### 3.5 Partial budget analysis

To evaluate the profitability of the treatment feeds partial budget analysis was conducted (Table 5). The results indicated that among all treatment groups, sheep supplemented with 75% TBDR and 25% FPH (*T*_4_) returned the highest NI (255.8. Ethiopian birr (ETB)) as compared to the other treatment groups and the control treatment. The control treatment had the lowest net return (82.7 ETB/sheep) compared to supplemented treatments.

**TABLE 5:** Partial budget analysis of Washera sheep fed on natural pasture grass hay and supplemented with a mixture of traditional brewery dried residue (TBDR) and field pea hull (FPH).

	Treatments			
Parameters	*T*_1_	*T*_2_	*T*_3_	*T*_4_
Sheep purchasing price (ETB)	1080	1080	1080	1080
Total basal diet intake (kg)	58.95	40.28	39.12	43.64
Total concentrate intake (kg)	4.5	45	45	45
**Feed cost**				
Total basal diet cost (ETB)	235.8	161.1	156.5	174.5
Total concentrate mix cost (ETB)	31.5	112.5	121.9	119.7
Total feed cost (TVC) (ETB)	267.3	273.6	278.4	294.2
Sheep selling price (ETB)	1430	1510	1570	1630
Total return (ETB)	350	430	490	550
Net return (ETB)	82.7	156.4	211.6	255.8
ΔNR	–	73.7	128.9	173.1
ΔTVC (ETB)	–	6.3	11.1	26.9
MRR%		1169	1161	643.5

*Note*: *T*_1_: hay + 50 g GNC; *T*_2_: *T*_1_ + 25% TBDR + 75% FPH; *T*_3_: *T*_1_ + 50% TBDR + 50% FPH; *T*_4_: *T*_1_ + 75% TBDR + 25% FPH.

Abbreviations: ΔNI, change in net income; ΔTVC, change in total variable cost; ETB, Ethiopian birr; GNC, ground nut cake; MRR, marginal return rate.

## 4 DISCUSSION

### 4.1 Chemical composition of treatment feeds

Dietary nutrients, especially energy and protein, are the major factors affecting the productivity of sheep. The lowest protein content in feed at which the sheep does not lose weight is above 7.5% in DM (Van Soest, 1994). Hence, the observed CP content of natural pasture hay used in the current study was below the maintenance requirement of sheep. Consequently, a 50 g/day/sheep GNC supplement was given to all sheep to satisfy the maintenance requirement of sheep in the control treatment. The NDF and ADF contents were also higher than the level (55%) above which voluntary feed intake is limited (Van Soest, 1994), indicating a need for supplement feeds to complement this feedstuff. The low nutrient content of natural pasture hay might be due to poor species composition of natural pasture hay (McDonald et al., 2010). The fact that, the lower CP and higher cell wall fibre content in hay refusals as compared to the offered might be associated with the selective feeding of sheep for more palatable and nutritious parts of hay than the unpalatable and lignified parts of it.

The CP content of FPH in this study was higher than the values of 10.5% and 8%, reported by Mesganaw (2014) and Mekasha et al. (2002), respectively. On the other hand, comparable NDF but lower ADF contents of FPH were recorded in the current study as compared to the report of Mekasha et al. (2002). The dissimilarity in the nutrient contents of FPH used in this study from the CP content of FPH used in the former studies might be due to the method of processing and variety of the field pea. The CP content of TBDR in the current study was comparable with the results of 21.2%, 22.3% and 21.8% reported by Solomon (2007), Ayenew et al. (2012), and Hagos (2014), respectively, but it was higher than the value 10.2% reported by Geaush (2011). Lower NDF content of TBDR in the current study was recorded than reported by Mekasha et al. (2002) and Hagos (2014), but it was comparable to 34.7% reported by Ayenew et al. (2012). Differences in NDF content of TBDR among studies might be associated with differences in the ingredients used to Tella preparation and the preparation methods followed in different areas of the country.

### 4.2 Feed intake

The DM intake of roughage feeds is determined by rumen fill and digesta passage rate, whereas the consumption of highly digestible, high‐energy (often low‐fibre, high‐concentrate) diets is controlled by the animal's energy demands and by metabolic factors (NRC (National Research Council), 2000). Hence, the lower total DM intake observed in the control treatment (*T*_1_) than the supplemented groups might be due to gut fill and lower digesta passage rate arising from deficiency of nutrients for rumen microorganisms. Grovum and William (1977) reported that longer feed retention in the rumen resulted in reduced feed intake. On the other hand, the improvement in total DM intake in the supplemented sheep, in general, could be attributed to a better supply of CP that promoted the proliferation of rumen microorganisms enabling more efficient digestion of the fibre components leading to more feed intake (McDonald et al., 2010). Generally, the total DM intake as a percentage of BW in the present study was within the range of 2%–6% recommended by the ARC (Agricultural Research Council) (1980) and 3.2% by Ranjhan (1997).

It was noted in previous studies that supplementation reduced the DM intake of hay per day (Ayele et al., 2017, 2022; Fentie & Solomon, 2008). It could be due to supplementation resulting in substitution rather than complementation of hay intake. ARC (Agricultural Research Council) (1980) also indicated that concentrate supplements increase total DM intake but reduce the intake of roughages due to substitution. The highest NDF and ADF intakes in sheep fed *T*_2_ as compared to the others and higher NDF and ADF intakes in *T*_2_ than *T*_3_ and *T*_4_ supplemented groups could be explained as a high content of these components in FPH as it was 75% in composition in *T*_2_ and partly due to function of high DM intake as compared to the control.

The levels of energy and protein requirements for maintenance and growth of sheep were estimated by different authors, and they indicated that a daily energy requirement for 20 kg sheep for maintenance is 1.17 Mcal digestible energy (NRC (National Research Council), 1981) and 30 g/day CP (ARC (Agricultural Research Council), 1980). According to ARC (Agricultural Research Council) (1980), a 20 kg sheep to gain a daily body weight of 50 g and 100 g required metabolizable energy of 5.1 and 6.2 MJ/day and 50 and 60 g/day CP, respectively. Accordingly, the daily CP intakes in the range of 118–135 g/day and ME intake in the range of 9.8–11 MJ/day in experimental sheep in the present study could support more than 100 g daily gain for 20 kg growing sheep.

### 4.3 Apparent digestibility of treatment feeds

The digestibility of a feedstuff is the proportion of the feed or any single nutrient of the feed which is not recovered in faeces. It is the single most important variable determining animal performance and voluntary intake, and it correlates with the amount of nutrients that can be extracted from feed. The digestibility of a feed is closely related to its chemical composition, especially the fibre fraction of a food has the greatest influence on its digestibility, and both the quantity and quality of the fibre are important (McDonald et al., 2010). Hence, the lowest DM, OM and CP digestibility recorded in sheep fed *T*_2_ supplement might be due to the high NDF content of the supplement.

Supplementing forage with a concentrate containing a readily available source of carbohydrates reduces NDF and ADF digestion due to its rapid fermentation to volatile fatty acids, which depresses the rumen pH to 6 or less. The low rumen pH inhibits cellulolytic microorganisms’ activity, and hence, fibre digestion reduces (McDonald et al., 2010). However, in the current study, this would not happen, but supplementation improved fibre digestion compared to the control and similar among supplement treatments. That might be partly explained by the supplements used that might contain a little amount of readily available carbohydrates and which might not bring rumen pH change but provides protein to enhance the activity of the rumen microorganisms, which are then better able to digest the fibre component of the feed (Ranjhan, 1997), and might be due to the high NDF and ADF content in FPH would aid in buffering, thus delaying any decrease in pH (Mekasha et al., 2002). Ranjhan (1997) and McDonald et al. (2002) indicated that protein‐rich feeds added to balance low protein roughages increase microbial activity in the rumen, consequently resulting in improved fermentation of the cell wall carbohydrates. The observed improvement in the apparent digestibility of DM and nutrients due to supplementation is in agreement with the report of Fentie and Solomon (2008), Ayele et al. (2017, 2022) in a trial using Farta sheep, three sheep breeds of Ethiopia, and the same sheep breed as the current study, respectively.

### 4.4 Body weight change and feed conversion efficiency

Body weight gain in animals is dependent on the animal's genetic potential and the extent to which the feed allows this potential to be expressed (Irshad et al., 2013; Oddy & Sainz, 2002), and it is an indicator of the potential of the feed along with the ability of the animals to extract nutrients from the feed. Protein and energy are the major nutrients affecting the weight gain of sheep (Freer & Dove, 2002). Accordingly, 22 g/day more ADG and higher FCE in sheep fed *T*_4_ (75% TBDR and 25% FPH) treatment than *T*_2_ and *T*_3_ in supplemented groups of sheep in this study could be attributed to the higher CP and ME contents of the supplement that promote more microbial population growth and therefore encourage digestion making nutrient available to increase weight gain in sheep.

The daily BW gain in *T*_4_ (79 g/day) of supplemented sheep in this study was similar to 78.7 g/day for the same breed of sheep supplemented with commercial concentrate mixed with the sweet blue lupine seed at various ratios (Likawent et al., 2012). Ayele et al. (2022) reported comparable ADG (84 g/day) for the same breed of sheep fed finger millet straw ad libitum + 280 g CM + 120 g grass pea hull. Similarly, comparable to the current result, 74 g/day ADG was reported for Farta sheep when sheep were supplemented with natural pasture hay + 150 g/day dried Atella + 100 g/day wheat bran (Teshager et al., 2022). On the other hand, the ADG values (56–79 g/day) in the current study for the supplemented groups of sheep were higher than 16–25 g/day observed in Washera sheep supplemented with 300 g/day of different forms of white lupin to Rhodes grass hay basal diet (Gebru et al., 2015). Growth rates between 45 and 64 (Mesganaw, 2014)) and 40–49 g/day (Assefu, 2012) have also been reported for Washera sheep fed hay as a basal diet and supplemented with FPH to concentrate ratio and roughage to concentrate ratio, respectively. However, the ADG values recorded from the current study as well as previous studies of Ethiopian sheep were much lower than the ADG values (256–290 g/day) recorded for Najdi lambs in Riyadh, Saud Arabia fed complete pelleted diets supplemented with different levels of *Arthrospira platensis* supplement (Alghonaim et al., 2022). Differences in ADG among studies could be attributed to variations in the supplements (type and amount), basal diets used, and the environment in which the experiments were conducted, and variations in sheep breeds used. Sheep in the control treatment in the current study were able to gain body weight, contrary to the results of Ayele et al. (2022) and Ayenew et al. (2012), who reported 5.6 and 23 g/day BW loss, and even better BW gain was observed in this study for the sheep in control treatment than that reported by Likawent et al. (2012) for the same breed of sheep. The better performance of sheep in the control treatment in this study could be attributed to the higher CP content of the GNC that was given to all sheep to fulfil the maintenance requirement of sheep in the control treatment, and it was above maintenance requirement.

### 4.5 Partial budget analysis

The fact that sheep fed *T*_4_ (75% TBDR and 25% FPH) fetched better price as compared to the others was due to higher final body weight of sheep which resulted in good body condition and attract the buyers. It is generally noted that animal feed cost is the single largest expense in animal production and shows feasibility of livestock enterprise. Therefore, any significant reduction in the cost of feeds will significantly reduce the overall cost of production and increase the profit margin of the farm. Thus, the results of this study suggested that *T*_4_ supplementation is potentially more profitable and economically beneficial than the other levels of supplementation.

## 5 CONCLUSION

The results of this study indicated that supplementation of poor quality diet like NPGH with the non‐conventional feeds (FPH and TBDR) improved both total DM, OM, CP and ME intakes and DM and other nutrients digestibility, which in turn resulted in improvement in ADG. Thus, based on the highest weight gain and net return a combination of the two supplements (75% TBDR and 25% FPH) as in *T*_4_ appeared to be recommendable for smallholder poor farmers in anywhere.

## AUTHOR CONTRIBUTIONS

The authors, Ayalew Abebe and Shashie Ayele Yimenu, contributed to research proposal writing, data collection, data analysis, data interpretation and draft document preparation. Shashie Ayele Yimenu prepared the manuscript for journal and all authors read and approved the final manuscript.

### ETHICS STATEMENT

Though our universities have no ethical approval committee, all animal handling practices followed the international guiding principles listed by the Council for International Organizations of Medical Sciences and the International Council for Laboratory Animal Science (2012).

### PEER REVIEW

The peer review history for this article is available at https://publons.com/publon/10.1002/vms3.1226.

## Data Availability

Data available on request from the authors.

## References

[vms31226-bib-0001] AFRC (Agricultural Food and Research Council) . (1993). Energy and protein requirements of ruminants. An advisory manual prepared by the agricultural food and research council technical committee on responses to nutrients. CAB International.

[vms31226-bib-0002] AOAC (Association of Official Analytical Chemists) . (1990). Official methods of analysis (15th ed). AOAC.

[vms31226-bib-0003] ARC (Agricultural Research Council) . (1980). The nutrient requirements of ruminant livestock. Technical review by an Agricultural Research Council Working Party, Common wealth Agricultural Bureau, Farnham Royal, UK. ARC (Agricultural Research Council).

[vms31226-bib-0004] Assefu, G. (2012). Comparative feedlot performance of washera and Horro sheep fed different roughage to concentrate ratio (An MSc Thesis). School of Animal and Range Sciences, Haramaya University.

[vms31226-bib-0005] Ayele, S. , & Urge, M. (2019). Productive and reproductive performances of indigenous sheep in Ethiopia: A review. Open Journal of Animal Sciences, 9, 97–120.

[vms31226-bib-0006] Ayele, S. , Urge, M. , Animut, G. , & Yusuf, M. (2017). Feed intake, digestibility, growth performance and blood profiles of three Ethiopian fat tail hair sheep fed hay supplemented with two levels of concentrate supplement. Open Journal of Animal Science, 7, 149–167.

[vms31226-bib-0007] Ayele, S. , Kefale, S. , & Addis, M. (2022). Effects of supplementing finger millet (*Eleucine coracana*) straw with grass pea (*Lathyrus sativus* L.) hulls and concentrate mixture on feed intake, digestibility and body weight change in Washera sheep. Open Journal of Animal Sciences, 12, 76–90.

[vms31226-bib-0008] Ayenew, A. , Berhan, T. , & Solomon, M. (2012). Feed Intake, digestibility and live weight change of lambs fed finger millet (*Eleusine coracana*) straw supplemented with atella, noug seed (*Guizotia abyssinica*) cake and their mixtures. Agricultura Tropica et Subtropica, 45(3), 105–111.

[vms31226-bib-0009] Council for international organizations of medical sciences and the international council for laboratory animal science. International guiding principles for biomedical research involving animals . (2012). http://grants.nih.gov/grants/olaw/Guiding_Principles_2012.pdf

[vms31226-bib-0010] CSA (Central Statistical Agency) . (2021). Livestock and livestock characteristics central statistical agency, Addis Ababa, Ethiopia. Statistical bulletin, II. CSA (Central Statistical Agency).

[vms31226-bib-0011] Derejaw, F. , Bekabil, F. , & Wagayehu, B. (2013). Determinants of the use of soil conservation technologies by small holder farmers: The case of Hulet Eju Enessie district, East Gojjam Zone, Ethiopia. Asian Journal of Agriculture and Food Sciences, 1(4), 123–129.

[vms31226-bib-0012] Ethiopia Sheep and Goat Productivity Improvement Program (ESGPIP) . (2009). Estimation of weight and age of sheep and goats. Technical bulletin, No. 23. Ethiopia Sheep and Goat Productivity Improvement Program (ESGPIP).

[vms31226-bib-0013] ESGPIP (Ethiopian Sheep & Goat Productivity Improvement Program) . (2011). Export requirements for meat and live small ruminants: How can development agents assist producers to improve small ruminant export? Technical bulletin, 47, Addis Abeba, Ethiopia. ESGPIP (Ethiopian Sheep & Goat Productivity Improvement Program).

[vms31226-bib-0014] FAO (Food and Agricultural Organization) . (2019). The future of livestock in Ethiopia. Opportunities and challenges in the face of uncertainty. Technical bulletin, (pp. 48). FAO (Food and Agricultural Organization).

[vms31226-bib-0015] Fentie, B. , & Solomon, M. (2008). Effects of supplementation of Farta sheep fed hay with sole or mixtures of noug seed meal and wheat bran on feed intake, digestibility and body weight change. Tropical Animal Health Production, 40(8), 597–606. 18975124 10.1007/s11250-008-9138-1

[vms31226-bib-0016] Freer, M. , & Dove, H. (2002). Sheep nutrition. CABI Publishing in Association with CSIRO Publishing.

[vms31226-bib-0017] Geaush, F. (2011). Comparison of supplementing urea molasses and urea Attela blocks on feed intake, digestibility, body weight change and carcass characteristics of male Black Head Ogaden sheep fed natural pasture hay (An MSc Thesis). School of Animal and Range Sciences, Haramaya University.

[vms31226-bib-0018] Gebru, T. , Firew, T. , Atsushi, T. , Yeshambel, M. , & Solomon, M. (2015). Effects of different forms of white lupin (*Lupinus albus*) grain supplementation on feed intake, digestibility, growth performance and carcass characteristics of Washera sheep fed Rhodes grass (*Chloris gayana*) hay‐based diet. Tropical Animal Health Production, 47, 1581–1590. 26250152 10.1007/s11250-015-0901-9

[vms31226-bib-0019] Grovum, W. L. , & William, J. V. (1977). Rate of passage of digesta in sheep. The effect of food intake on mathematical prediction of the kinetics of digesta in the reticulo‐rumen and intestines. British Journal of Nutrition, 38, 425–436. 588540 10.1079/bjn19770107

[vms31226-bib-0020] Hagos, H. (2014). *Effect of supplementation of concentrate mixture, dried local brewery byproduct (Atella), Faidherbia* albida *and* Sesbania sesban *on the performance of local sheep fed hay basal diet* (An MSc Thesis). School of Animal and Range Sciences, Haramaya University.

[vms31226-bib-0021] Irshad, A. , Kandeepan, G. , Kumar, S. , Ashish, A. , Kumar, M. , Vishnuraj, R. , & Shukla, V. (2013). Factors influencing carcass composition of livestock: A review. Journal of Animal Production Advance, 3(5), 177–186.

[vms31226-bib-0022] Likawent, Y. , Claudia, K. , Firew, T. , & Kurt, J. P (2012). Sweet blue lupin (*Lupinus angustifolius L*.) seed as a substitute for concentrate mix supplement in the diets of yearling Washera rams fed on natural pasture hay as basal diet in Ethiopia. Tropical Animal Health Production, 44, 1255–1261. 22228539 10.1007/s11250-011-0066-0

[vms31226-bib-0023] McDonald, P. , Edwards, R. A. , Greenhalgh, J. F. D. , Morgan, C. A. , Sinclair, L. A. , & Wilkinson, R. G. (2010). Animal nutrition (7th ed.). Prentice Hall, Pearson.

[vms31226-bib-0024] McDonald, P. , Edwards, R. A. , Greenhalgh, J. F. D. , & Morgan, C. A. (2002). Animal nutrition (6th ed.). Prentice Hall.

[vms31226-bib-0025] McKay, K. , Schatz, B. , & Endres, G. (2003). Field pea production. Technical bulletin (pp. 58105). North Dakota State University.

[vms31226-bib-0026] Mekasha, Y. , Tegegn, A. , Yami, A. , & Umunna, N. N. (2002). Evaluation of non‐conventional agro‐industrial by‐products as supplementary feeds for ruminants: *In vitro* and metabolism study with sheep. Small Ruminant Research, 44, 25–35.

[vms31226-bib-0027] Mekcha, E. , Alemu, B. , & Ayele, S. (2019). Effect of *Atella, Birint* and their mixture supplementation on feed intake, digestibility and body weight change of Washera sheep fed oat (*Avena sativa*) straw basal diet, in Machackel district, East Gojjam Zone. Journal of Agricultural Economics & Rural Development, 5(1), 555–562.

[vms31226-bib-0028] Mesganaw, A. (2014). *Effect of different proportions of field pea (Pisum* sativum) hulls and concentrate mixture supplementation on feed intake, digestibility, and body weight *change and carcass parameters of Washera sheep fed a basal diet of grass hay* (MSc Thesis). School of Animal and Range Sciences, Haramaya University.

[vms31226-bib-0029] Panadi, M. , Mat, K. , Rahman, M. M. , Khan, M. A. K. G. , Balakrishnan, M. , & Rusli, N. D. (2021). Nutrient intake, growth performance and nutrient digestibility of pre and post‐weaning Dorper lambs fed varying crude protein level. Tropical Animal Health and Production, 53, 515. 10.1007/s11250-021-02953-3 34647184

[vms31226-bib-0030] Alghonaim, A. A. , Alqahtani, M. F. , Al‐Garadi, M. A. , Suliman, G. M. , Al‐Baadani, H. H. , AL‐Badwi, M. A. , Abdelrahman, M. M. , Alowaimer, A. N. , Khan, R. U. , & Alhidary, I. A. (2022). Effects of different levels of spirulina (*Arthrospira platensis*) supplementation on productive performance, nutrient digestibility, blood metabolites, and meat quality of growing Najdi lambs. Tropical Animal Health and Production, 54, 124. 10.1007/s11250-022-03115-9 35235076

[vms31226-bib-0031] Nigussie, H. , Mekasha, Y. , & Kebede, K. (2013). Production objectives, breeding practices and selection criteria of indigenous sheep in eastern Ethiopia. LRRD, 25(9).

[vms31226-bib-0032] NRC (National Research Council) . (1981). Nutrient requirements of domestic animals. National Academy of Sciences—National Research Council.

[vms31226-bib-0033] NRC (National Research Council) . (2000). Nutrient requirements of beef cattle. (7th revised ed.). National Academy Press.

[vms31226-bib-0034] Oddy, V. H. , & Sainz, R. D. (2002). Nutrition for sheep‐meat production In M. Freer & H. Dove (Eds.), CSIRO plant industry Canberra Australia: Sheep nutrition. CABI Publishing, CAB International.

[vms31226-bib-0035] Ranjhan, S. K. (1997). Animal nutrition in the tropics (4th ed.). Vikas Publishing House Pvt Ltd.

[vms31226-bib-0036] SAS (Statistical Analysis Systems Institute) . (2008). Version 9.1. SAS Institute Inc.

[vms31226-bib-0037] Solomon, D. (2007). Comparative nutritive value of *Atella* and industrial brewers grains in chicken starter ration in Ethiopia. Livestock Research for Rural Development (LRRD), 19(1).

[vms31226-bib-0038] Teshager, D. , Mekuriaw, Y. , & Beyero, N. (2022). Protein supplement potential of *Dodonaea angustifolia* leaves by replacing atella on nutrient utilization and performance of Farta sheep fed natural pasture hay basal diet. Veterinary Medicine and Science, 8, 2230–2240. 10.1002/vms3.898 PMC9514476 35994299

[vms31226-bib-0039] Upton, M. (1979). Farm management in Africa: The principle of production and planning. Oxford University press.

[vms31226-bib-0040] Van Soest, P. J. , & Robertson, J. B. (1985). Analysis of forages and fibrous foods: A laboratory manual for animal science. Cornell University.

[vms31226-bib-0041] Van Soest, P. J. (1994). Nutritional ecology of ruminants (2nd ed.). Cornell University Press.

[vms31226-bib-0042] Zewdu, E. , Aynalem, H. , Markos, T. , Sharma, A. K. , Dejene, A. , Sölkner, J. , & Maria, W. (2009). Morphological characterization of Bonga and Horro indigenous sheep breeds under smallholder conditions in Ethiopia. Ethiopian Journal Animal Production, 9, 117–136.

